# Impact of stressful life events on central adiposity in the Pelotas Birth Cohort

**DOI:** 10.11606/S1518-8787.2018052000161

**Published:** 2018-05-07

**Authors:** Pamela J Surkan, Kwame S Sakyi, Alice Hu, Maria T Olinto, Helen Gonçalves, Bernardo L Horta, Denise P Gigante

**Affiliations:** I Johns Hopkins School of Public Health. Department of International Health. Social and Behavioral Interventions Program. Baltimore, Maryland, USA; II Oakland University. School of Health Sciences. Department of Public and Environmental Wellness. Rochester, Michigan, USA; III Universidade do Vale do Rio dos Sinos. Programa de Pós-Graduação em Saúde Coletiva. São Leopoldo, RS, Brasil; IV Federal University of Health Science of Porto Alegre. Department of Nutrition. Porto Alegre, RS, Brasil; V Universidade Federal de Pelotas. Programa de Pós-Graduação em Epidemiologia. Pelotas, RS, Brasil

**Keywords:** Stress, Psychological, Life Change Events, Waist-Hip Ratio, utilization, Weight Gain, Gender and Health

## Abstract

**OBJECTIVE:**

To investigate how stressful life events and social support relate to central adiposity in Southern Brazil.

**METHODS:**

Data included information from 802 participants in the 1982 Pelotas Birth Cohort that was collect in 2004-2005 and 2006. Stratifying by sex, we studied self-reported stressful life events during the year before 2004-2005 in relation to change in waist circumference between 2004-2005 and 2006 and waist-to-hip ratio in 2006, using both bivariate and multivariate linear regression models.

**RESULTS:**

In adjusted models, the experience of stressful life events during the year before 2004-2005 predicted a change in waist circumference in 2006 in men and a change in both waist-to-hip ratio in 2006 and waist circumference between 2004-2005 and 2006 in women. Men who experienced two or more stressful events had on average a one centimeter increase in their waist circumference between 2004-2005 and 2006 (β = 0.97, 95%CI 0.02-1.92), compared to those reporting no stressful events. For women, those who had one and those who had two or more stressful life events had over a 1 cm increase in their waist circumference from 2004-2005 to 2006 (β = 1.37, 95%CI 0.17-2.54; β = 1.26, 95%CI 0.11-2.40, respectively), compared to those who did not experience any stressful event. For both sexes, social support level was not significantly related to either waist-to-hip ratio or change in waist circumference, and it did not modify the association between stress and central adiposity.

**CONCLUSIONS:**

The experience of more than one stressful life event was associated with distinct indicators of central adiposity for men *versus* women.

## INTRODUCTION

Central adiposity, otherwise known as abdominal obesity, is a risk factor for coronary heart disease, stroke, and diabetes, and is characterized by the distribution of visceral fat in the abdominal area ^1^ ^,^ ^2^. A variety of complex mechanisms and hypotheses have been postulated (e.g., the portal hypothesis) regarding pathways through which visceral fat can lead to insulin resistance ^3^. Body mass index (BMI) is a poor predictor of cardiovascular disease compared to measures of central adiposity, such as waist-to-hip ratio (WHR) and waist circumference (WC)^4^. Interestingly, however, a very high correlation has been observed between WC and BMI among Brazilians^5^.

Epidemiological studies have shown a relation between social class and central adiposity^6^, with work-related or other stressors implicated in its development^6^ ^-^ ^8^; specifically, stress activates the hypothalamic-pituitary-adrenal axis and sympathetic nervous system, which leads to hormonal abnormalities including increased cortisol secretion and development of visceral fat^9^. Chronic stress may play a role in the development of fat from another pathway, from its influence on dietary intake, including the consumption of energy-dense foods^8^ ^,^ ^10^ ^,^ ^11^.

Psychosocial stress derived from the social environment, e.g., loss of a family member or financial difficulties, is thought to have a cumulative effect on physical and mental health^12^. Although a relationship between stress and central adiposity has been observed, the types of psychosocial stressors that may be important and the role of social support systems across different cultures have not been elucidated. According to the stress-buffering hypothesis, social support may be protective against the adverse effects of stress^13^. We hypothesized that stressful events would be positively associated with central adiposity, while social support would be inversely associated with it. Additionally, we hypothesized that high levels of social support may serve as a buffer for the relationship between stressful events and central adiposity. The main aims of our study were to investigate how stressful life events and how social support relate to central adiposity, using a cohort study from Southern Brazil. The secondary aim was to assess whether social support buffered the impact of stress on central adiposity by examining the effect modification by social support.

## METHODS

This study was based on the follow-up data from the Pelotas Birth Cohort carried out in Southern Brazil from October 2004 to August 2005 and from January to April 2006. The study population consisted of 802 adults (416 men and 386 women) for whom follow-up data on WC was available. All pregnant women were excluded from this study.

### Study Procedures

The study began in 1982 and has continued to follow 5,914 live births in the three maternities from Pelotas, representing 99.2% of all births whose families lived in the urban area of the city^14^ ^,^ ^15^. A range of information including demographic, socioeconomic, and anthropometric data were collected, as well as data on health-related variables^15^. In the 2004-2005 visit, trained field workers interviewed 4,297 participants, representing a follow-up rate of 77.4% when added to the 282 individuals known to have died. Weight circumference was assessed in a subsample of 856 participants^16^, corresponding to a random sample of census tracks in the city. Our 2006 follow-up consisted of 802 adults, a subset of the 856 individuals with information on WC. Details about data quality control with scale calibration, standardization sessions, and double data entry have been reported previously^15^.

### Measures

Our primary outcomes of interest were 1) change in WC from 2004-2005 to 2006 and 2) WHR in 2006. Trained interviewers collected WC and hip conference, which were used to determine WHR (WC divided by hip conference). Weight circumference, an indicator of central adiposity, was measured in centimeters at the narrowest part of the trunk, right above the navel at both time points^17^ ^,^ ^18^. Quality control was checked by a supervisor repeating 10% of the measurements, showing over 95% repeatability.

Our main independent variable included six questions to assess exposure to stressful life events within the last year before the interview in 2004-2005 asking whether the respondent: 1) had some type of health problem that did not allow for normal activities, 2) experienced the death of a close family member, 3) had a serious relationship end (with a partner or divorce), 4) faced financial difficulties that were more serious than usual, 5) had to move out of the house against his/her will, 6) had nervous and emotional problems. Items representing stressful events were selected by a team led by a Brazilian anthropologist (co-author HG) adapted from other existing scales^19^ ^-^ ^22^ and were deemed culturally relevant stressors in the local setting of Southern Brazil. Each event was given one point. Stress score was categorized as none (score = 0), low (1-2), or high (> 2).

Social support (high *versus* low or none) was measured by two questions assessing the presence of material (i.e., monetary) and emotional support from family and friends, respectively. We considered the lack of either or both types of support as low or no social support. Other variables considered were: education (≤ 8, > 8 years), parity (no children, ≥ 1 child), race (white, non-white), household income tertile (low, medium, high), alcohol intake (≤ 2, > 2 drinks per week), smoking status (never, former, current), and BMI in 2004, calculated with standard units of kg/m2 and categorized using the WHO cut-off value for overweight: < 25 *versus* ≥ 25^23^.

### Statistical Analysis

All statistical analyses were stratified by sex, as abdominal fat patterns differ between men and women9. One-way ANOVA was used to test sex differences for continuous variables (WC and WHR), chi-square tests were used to assess sex differences in categorical variables, and t-tests of proportions were used to evaluate sex differences by stress score. Quantile-quantile plots and Shapiro-Wilk test were used to check for a normal distribution of the difference in WC. Leverage-*versus*-residual squared plots and box plots were fit to identify points with higher than average leverage or residuals. Observations with high leverage or residuals were dropped. A total of 16 observations (14 for men and 2 for women) were excluded from the analysis.

In the bivariate analyses, beta coefficients and 95% confidence intervals were calculated for each of the predictors in relation to WHR in 2006, change in WC between 2004-2005 and 2006, and covariates measured in 2004-2005. Multiple regression analyses were also performed using WHR and change in WC as outcomes and the social support (high *versus* low or none) and stressful life events categories (none, one, or two or more) as independent variables, while stratified by sex. Covariate selection was based on factors important for central adiposity based on the current literature (household income, education, alcohol intake, smoking status, race, and parity) or on factors that had p-value < 0.20 in the bivariate analyses. We also controlled for 2004 BMI in all analyses with change in WC as outcome. For linear regression models, the normality assumption (of residuals) was checked using quantile-quantile plots. We created an interaction term between stressful life events and social support and performed the Wald test to determine if the relation between stressful life events and central adiposity was modified by social support for either sex (p < 0.05).

Data were analyzed using Stata statistical software version 11.0 (StataCorp, 2009, Version 11, College Station, Texas, USA).

### Ethics

All individuals provided written informed consent in both the 2004-2005 and 2006 waves of data collection. The study was approved by the Human Subjects Committee of the Universidade Federal de Pelotas.

## RESULTS

### Descriptive Characteristics of Study Population and Stressful Events in Men and Women

In terms of stressful life events, women reported both more serious health problems (41% women; 29% men) and more emotional problems (35% women; 21% men) within the last year. Other individual stressful life event items were not significantly different when comparing sexes (Table 1). This is consistent with the fact that, when the scale was used as a whole, women were also more likely to report having experienced two or more stressful life events in the last year (47%) compared to men (36%) (Table 2).

**Table 1:** Stressful life events assessed in 2004-2005, stratified by sex.

Stressful life events	Total	Men	Women	p
	(n = 802)	(n = 416)	(n = 386)	
	n (%)	n (%)	n (%)	
Serious health problem	277 (34.5)	120 (28.9)	157 (40.7)	< 0.001
Death of relative	215 (26.8)	102 (24.5)	113 (29.3)	0.13
End of a serious relationship/Divorce	150 (18.7)	74 (17.8)	76 (19.7)	0.49
Financial difficulties	195 (24.3)	102 (24.5)	93 (24.1)	0.89
Eviction from home	43 (5.4)	18 (4.3)	25 (6.5)	0.18
Emotional problems	220 (27.4)	86 (20.7)	134 (34.7)	< 0.001

**Table 2:** Descriptive characteristics of adults born in 1982, stratified by sex.

Categorical variables		Total	Males	Females	p
		(n = 802)	(n = 416)	(n = 386)	
		n (%)	n (%)	n (%)	
Stressful life events					0.002
	None (0)	216 (26.9)	129 (31.0)	87 (22.5)	
	One (1)	257 (32.0)	139 (33.4)	118 (30.6)	
	Two or more (≥ 2)	329 (41.0)	148 (35.6)	181 (46.9)	
Social support					0.20
	High	625 (78.0)	317 (76.2)	308 (80.0)	
	Low or none	176 (22.0)	99 (23.8)	77 (20.0)	
Education (years)					< 0.001
	≤ 8	230 (28.7)	143 (34.4)	87 (22.5)	
	> 8	572 (71.3)	273 (65.6)	299 (77.5)	
Parity					< 0.001
	No children	561 (70.0)	241 (62.4)	320 (76.9)	
	≥ 1	241 (30.1)	145 (37.6)	96 (23.1)	
Race					0.16
	White	626 (78.3)	322 (77.8)	314 (79.0)	
	Non-white*	173 (21.6)	92(22.2)	81 (21.0)	
Household income (tertiles)					0.34
	Low	254 (33.5)	124 (31.2)	130 (35.9)	
	Medium	257 (33.9)	142 (35.8)	115 (31.8)	
	High	248 (32.7)	117 (32.3)	131 (33.0)	
Alcohol intake (per week)					< 0.001
	≤ 2 drinks	538 (67.1)	238 (57.2)	300 (77.7)	
	> 2 drinks	264 (32.9)	178 (42.8)	86 (22.3)	
Smoking status					0.47
	Never	556 (69.3)	281 (67.5)	275 (71.2)	
	Former	63 (7.9)	33 (7.9)	30 (7.8)	
	Current	183 (22.8)	102 (24.5)	81 (21.0)	
BMI in 2004					0.13
	< 25	570 (71.1)	286 (73.6)	284(68.7)	
	≥ 25	232 (28.9)	102 (31.2)	130 (26.4)	
Continuous variables		Mean (SD)	Mean (SD)	Mean (SD)	p
Waist-hip ratio		0.80 (0.07)	0.84 (0.05)	0.75 (0.05)	< 0.001
Change in waist circumference between 2004 and 2006 (cm)		0.44 (0.18)	1.29 (0.25)	–0.48 (0.25)	< 0.001

BMI: body mass index

* Almost all individuals in this category were black or mixed race, with the exception of four who identified as Asian and two who identified as indigenous.

Most other variables included in the model also differed between sexes, with more women having higher educational attainment but being less likely to have at least one child and to have > 2 drinks per week (Table 2). Males on average had an increase in WC (1.29 cm), while females had overall decrease (-0.48 cm) in WC between 2004 and 2006. As expected, males had on average a higher mean WHR (0.84 cm) compared to women (0.75 cm) (Table 2). There was no difference between men and women in social support, race, household income, smoking status, or BMI in 2004 (Table 2).

### Stressful Events and Measures of Central Adiposity in Men

Crude analyses in males showed only two statistically significant associations with indicators of central adiposity; men who had BMI ≥ 25 in 2004-2005 were more likely to have a higher WHR in 2006 (β = 0.05, 95%CI 0.04-0.06). Similarly, higher WC in men in 2004-2005 was also associated with a higher WHR in 2006 (β = 0.003, 95%CI 0.002-0.003). Men with more than eight years of education were more likely to have an increase in their WC from 2004-2005 to 2006 (β = 0.98, 95%CI 0.17-1.79) (Table 3).

**Table 3:** Crude analyses of the demographic information, stressful life events, and social support in relation to measures of central adiposity in adults born in 1982, stratified by sex.

Variables		Men				Women			
		Waist-to-hip ratio in 2006		Change in waist circumference between 2004-2005 and 2006		Waist-to-hip ratio in 2006		Change in waist circumference between 2004-2005 and 2006	
		β (95%CI)	p	β (95%CI)	p	β (95%CI)	p	β (95%CI)	p
Stressful life events									
	None	Ref		Ref		Ref		Ref	
	One	0.0008 (-0.01-0.01)	0.88	0.27 (-0.69-1.24)	0.57	**0.02 (0.008-0.04)**	**0.002**	0.72 (-0.58-2.03)	0.27
	Two or more	0.004 (-0.01-0.01)	0.51	0.93(-0.02-1.88)	0.06	**0.01 (0.009-0.03)**	**0.04**	0.75 (-0.44-1.96)	0.21
Social support									
	High	Ref		Ref		**Ref**		Ref	
	Low or none	0.002 (-0.009-0.01)	0.74	–0.80 (-1.71-0.10)	0.08	**0.01 (0.001-0.03)**	**0.04**	–0.16 (-1.33-1.01)	0.78
Education									
	≤ 8 years	Ref		Ref		**Ref**		Ref	
	> 8 years	–0.0002 (-0.01-0.01)	0.97	**0.98 (0.17-1.79)**	0.02	**–0.03 (-0.04--0.02)**	**< 0.001**	0.99 (-0.13-2.11)	0.08
Parity									
	No children	Ref		Ref		**Ref**		Ref	
	≥ 1 child	NA	NA	NA	NA	**0.03 (0.02-0.04)**	**< 0.001**	**–1.15 (-2.11--0.18)**	**0.02**
Race									
	White	Ref		Ref		**Ref**		Ref	
	Non-white	–0.002 (-0.01-0.01)	0.71	–0.02 (-1.03-1.00)	0.97	**0.02 (0.003-0.029)**	**0.02**	0.32 (-0.87-1.52)	0.60
Household income (tertiles)									
	Low	Ref		Ref		**Ref**		Ref	
	Medium	0.003(-0.008-0.01)	0.65	0.31 (-0.64-1.27)	0.52	**–0.02 (-0.03--0.005)**	**0.005**	0.60 (-0.54-1.74)	0.30
	High	0.01(-0.001-0.02)	0.08	0.50 (-0.47-1.46)	0.31	**–0.04 (-0.05--0.02)**	**< 0.001**	0.89 (-0.25-2.02)	0.13
Alcohol intake (per week)									
	≤ 2 drinks	Ref		Ref		**Ref**		Ref	
	> 2 drinks	0.001 (-0.01-0.003)	0.34	0.08 (-0.11-0.28)	0.37	0.006 (-0.007-0.02)	0.35	–0.01 (-1.15-1.11)	0.98
Smoking status									
	Never	Ref		Ref		Ref		Ref	
	Former	–0.005 (-0.02-0.01)	0.57	–0.09 (-1.53-1.41)	0.90	–0.00003 (-0.02-0.02)	0.99	–0.93 (-2.70-0.83)	0.31
	Current	0.003 (-0.01-0.01)	0.50	0.11 (-0.66-1.21)	0.81	**0.03 (0.01-0.04)**	**< 0.001**	–0.46 (-1.70-0.75)	0.44
BMI in 2004									
	< 25	Ref		Ref		Ref		Ref	
	≥ 25	**0.05 (0.04-0.06)**	**< 0.001**	0.42 (-0.42,1.25)	0.33	**0.05 (0.04-0.06)**	**< 0.001**	–1.06 (-2.12-0.13)	0.08
Waist circumference in 2004 (cm)		**0.003 (0.003-0.004)**	**< 0.001**	–	–	**0.003 (0.002-0.003)**	**< 0.001**	–	–

Ref: reference; BMI: body mass index; NA: Not applicable

For females, the sample size is 384 for all variables, except for race and social support, which had one missing value each.

For men, sample size is 406 for all variables, except for race (n = 388), which had 18 missing values.

Bold values indicate statistically significant results using a cut-off of p < 0.05.

For men, in the multivariable models adjusted for education, household income, alcohol intake, smoking status, BMI in 2004, and WC in 2004, we found a positive relation between having at least two stressful life events and increased WC between 2004-2005 and 2006 (β = 0.97, 95%CI 0.02-1.92). This association between stressful life events and change in WC in men was not modified by social support for men (Wald test, p = 0.77).

### Stressful Events and Measures of Central Adiposity in Women

For women, almost all factors were associated with WHR in 2006 in our crude analysis. Compared to women reporting no stressful life events in the last year, those reporting one (β = 0.02, 95%CI 0.008-0.04) or more than one (β = 0.01, 95%CI 0.009-0.03) had a significantly higher WHR in 2006. Low or no social support was associated with 0.01 cm increase in WC between 2004-2005 and 2006 (β = 0.01, 95%CI 0.001-0.03). In addition, having one or more children (β = 0.03, 95%CI 0.02-0.04), being a current smoker (β = 0.03, 95%CI 0.01-0.04), being non-white (β = 0.02, 95%CI 0.02-0.04), being overweight or obese in 2004 (β = 0.05, 95%CI 0.04-0.06), and having a higher WC in 2004-2005 (β = 0.003, 95%CI 0.002-0.003) were positively associated with WHR in 2006. On the other hand, compared to women with eight years of education or less, women with > 8 years of education in 2004-2005 had lower WHR in 2006 (β = −0.03, 95%CI −0.04- −0.02). Similarly, compared to those with the lowest income level, women from medium and high income level households had lower WHR in 2006 (β = −0.02, 95%CI −0.03- −0.005; β = −0.04, 95%CI −0.05- −0.02, respectively). Among the exposures and covariates assessed, only parity, i.e., having ever had at least one child, was associated with reduced WC (β = −1.15, 95%CI −2.11- −0.18) (Table 3).

In the multivariable models adjusted for the same covariates (education, race, household income, alcohol intake, smoking status, BMI in 2004-2005) in addition to parity in the analysis for women, we found a statistically significant positive association between having one stressful life event and WHR in 2006 (β = 0.02, 95%CI 0.003-0.03) (Table 4). Moreover, compared to women who reported no stressful life events in the past year, women who had one or more than one stressful life events had an increase in WC between 2004 and 2006 that were, on average, 1.37 cm or 1.26 cm greater, respectively, after holding the other covariates constant (one stressful life event: β = 1.37, 95%CI 0.17-2.54; two or more stressful life events: β = 1.26, 95%CI 0.11-2.40). No adjusted models assessing the association between social support and our indicators of central adiposity were significant for men or women (Table 4). Social support did not modify the relation between stressful life events and change in WC (2004-2006) (Wald test, p = 0.28) or WHR (Wald test, p = 0.63) in women.

**Table 4:** Adjusted analyses of stressful life events and social support variables in relation to measures of central adiposity in adults born in 1982, stratified by sex.

Variable		Men (n = 406)				Women (n = 377)			
		Waist-to-hip ratio in 2006		Change in waist circumference between 2004-2005 and 2006		Waist-to-hip ratio in 2006		Change in waist circumference between 2004-2005 and 2006	
		β (95%CI)	p	β (95%CI)	p	β (95%CI)	p	β (95%CI)	p
Adjusted models with stress									
Stressful life events									
	None	Ref		Ref		Ref		Ref	
	One	–0.001 (-0.01-0.008)	0.81	0.21 (-0.75-1.17)	0.66	**0.02 (0.003-0.03)**	**0.01**	**1.37 (0.17-2.54)**	**0.02**
	Two or more	0.003 (-0.006-0.013)	0.48	**0.97 (0.02-1.92)**	**0.045**	0.01 (-0.002-0.02)	0.10	**1.26 (0.11-2.40)**	**0.03**
Adjusted models with social support									
Social support									
	High	Ref		Ref		Ref		Ref	
	Low or none	–0.0004 (-0.01-0.01)	0.92	–0.64 (-1.55-0.28)	0.17	0.002 (-0.01-0.01)	0.29	0.36 (-0.76-1.49)	0.64

Models for waist-to-hip ratio are adjusted for education (> 8 versus ≤ 8 years), household income (tertiles), number of alcoholic drinks per week (> 2 versus ≤ 2), smoking status (never, former, current), and BMI in 2004 (< 25 versus ≥ 25). Models for change in waist circumference are adjusted for these variables, as well as waist circumference in 2004. Analyses for women were also adjusted for parity (no children versus ≥ 1 child) and race (white versus non-white).

Note: The models with stressful life events were not adjusted for the social support variable and the models with social support were not adjusted for stressful life events.

Bold values indicate statistically significant results using a cut-off of p < 0.05.

## DISCUSSION

We found that women reported a higher proportion of stressful life events than men, which appeared to be primarily due to the relative likelihood of more serious health and emotional problems. We also found that stressful life events were more related to measures of central adiposity in women than in men. Women who reported one stressful life event in 2004-2005 were significantly more likely to have higher WHR in 2006 and also experience an increase in change in WC over a one- to two-year period, compared to women reporting no stressful life events. Compared to the same group, those who reported two or more stressful life events had a statistically significant increase in WC from 2005 to 2006. In men, the experience of stressful life events resulted in an increase in WC but not in WHR, with those experiencing two or more stressful life events before 2004-2005 having approximately one centimeter increase in WC between 2004-2005 and 2006. While social support was associated with WHR in women, after adjustment for covariates, there was no clear relation between social support and WHR. In adjusted models, social support was not associated with either indicator of central adiposity in men or women.

Our study showed modest positive associations between increased stressful life events and change in WC in both men and women. Another study has found central fat distribution to mediate the association between stress eating in non-diabetics (defined as eating more or one's favorite foods because of stress) and measures of glucoregulation (e.g., glucose, insulin, insulin resistance)^24^. A British study that examined life events as predictors of WC has found illness (referring to disability in oneself or a child) to be related to increased WC^25^. Another stressor, interpersonal discrimination, has also been associated with increases in WC over a period of nine years in a US population, which was more pronounced in men than women^26^. A recent study from Britain has found the negative aspects of a close relationship to be related to increases in WC over a similar time period^27^. Nonetheless, other research found no evidence of significant associations between self-perceived stress, perceived life stress, and cortisol with WC^28^ ^,^ ^29^.

In terms of WHR, we found a positive significant association only in women and not men. Similar to our findings, higher perceived stress among a US sample of diabetic adults was found to be associated with WHR in women, but not men30. Two studies have shown null associations. In Finnish men, no relation was found between negative life changes (e.g., loss of job or increased conflicts with a spouse) and WHR31. Similarly, there were no significant changes in the association between life events and WHR among Dutch young adults of either sex32.

We found no relation between social support and WC or WHR in either women or men. Regarding the literature on social support and central adiposity, findings are mixed with some studies showing associations^33^ ^-^ ^35^, others reporting null findings^28^ ^,^ ^36^, or only associations among males^30^ ^,^ ^37^. In a small sample of women (n = 41), unadjusted comparisons suggested that women with normal WC had higher levels of social support than women with high WC^33^. These findings have been reinforced by larger studies indicating that the lower levels of perceived support in women were associated with WC and WHR after adjustment for BMI^34^ ^,^ ^38^. A study with adolescents has shown that those with less supportive relationships had increased WHR over a three-year period, adjusting for baseline WHR^35^. One of the few other studies in Latin America, with Bolivian women, has reported non-significant relations between instrumental social support and central adiposity, measured by the subscapular to triceps skinfold ratio^36^.

The strengths of our study include longitudinal follow-up of a relatively large cohort of men and women representative of a medium-sized city in southern Brazil. As weight gain or loss is typically gradual, the relatively short time of follow-up could be a limitation and a potential explanation for the relatively small changes observed. On the other hand, the biological and behavioral impacts of these life events are likely to be most relevant in the period immediately following the event^39^. Similarly, both our measures of stress and social support were limited by the number and types of stressors included in the data set. Although adapted from standard items of other validated instruments^19^ ^-^ ^22^, our scale had not been validated in Brazil. Given the Brazilian cultural context, it is possible that other types of life events not available in our data set may be more relevant to central adiposity.

Our study showed varied, modest associations between increased stressful life events and WC and WHR. Mechanisms that have been proposed include alterations caused by changes in the functioning of the hypothalamic-pituitary-adrenal axis^9^ and higher dietary intake of foods high in fat and sugar as a response to stress^10^. Given the inconsistencies in the literature to date^28^ ^,^ ^30^ ^,^ ^33^ ^-^ ^37^, further research is needed to confirm these findings and to examine how stressful events relate to change in WHR. We could not address this issue of change over time in this study because data were not available on WHR in 2004. Moreover, in future studies, it would be interesting to explore changes in WC adjusted for changes in BMI, i.e., to evaluate change in the degree of centrality. We could not assess this because we did not have measures on BMI in 2006. Furthermore, as a more precise measure than BMI in 2004, we would have liked to have been able to adjust for change in BMI between 2004 and 2006, given that variation in WC could have occurred because of a change in BMI that is not necessarily attributable to central adiposity. Future studies would benefit from including a wider range and a more extensive list of stressful events, which could be studied both individually and cumulatively.
